# The efficacy of hydrogel foams in talc Pleurodesis

**DOI:** 10.1186/s13019-020-01098-y

**Published:** 2020-04-15

**Authors:** Joy Baxter, Thamires A. Lima, Richard Huneke, Colin Joseph Kanach, Priya Johal, Emily Reimold, Nicolas J. Alvarez, Glenn W. Laub

**Affiliations:** 1 Department of Chemical and Biological Engineering, Drexel University, Philadelphia, PA USA; 2 Department of Microbiology and Immunology, Drexel University, Philadelphia, PA USA; 3 Department of Pathology, Drexel University College of Medicine, Philadelphia, PA 19129 USA; 4 ULAR, Drexel University, Philadelphia, PA USA; 5 Department of Cardiothoracic Surgery, Drexel University College of Medicine, Philadelphia, PA USA

**Keywords:** Talc pleurodesis, Foam delivery system, Adhesion formation, New Zealand rabbit model

## Abstract

**Background:**

Malignant pleural effusions are a serious complication of many late stage cancers that adversely affect quality of life. Pleurodesis with talc slurry is a standard treatment option, but clinical failures occur, possible due to poor talc delivery. A novel drug-delivery system was developed that fills the entire thoracic cavity with a liquid foam containing talc. The foam is designed to gel and adhere to the tissue walls at body temperature, to improve talc deposition and efficacy.

**Methods:**

Rheology, foam stability, and ex-vivo coating and bio-adhesion studies were performed on three concentrations of a novel hydrogel talc foam system that was developed to improve delivery of talc to the pleural surfaces. A New Zealand rabbit model of pleurodesis was used to evaluate effectiveness of the foams at inducing adhesion formation and compared to talc slurry. The rabbits were recovered after they had one of the test agents instilled into their pleura, and then sacrificed after 28 days. Pleurodesis was assessed by a blinded pathologist using a standardized pathological scoring system.

**Results:**

All talc foam formulations produced foams that gelled at physiological temperatures and were relatively stable for at least two hours. As the concentration of the formulation increased the gelation temperature decreased and the foam adhesiveness increased. Rabbits that received talc foam had significantly greater adhesion formation than talc slurry (mean score of 2.21 vs. 1.18 (*p* < 0.05)). Rabbits that received the 20% foam developed the most adhesions.

**Conclusions:**

This study demonstrates that our triblock copolymer hydrogel foam delivery system enhances adhesion formation in an experimental model. This novel approach can have important clinical impact, potentially improving efficacy of existing therapies and reducing the need for more invasive treatments.

## Background

Malignant pleural effusions (MPEs) occur in approximately 200,000 patients in the United States each year, affecting between 7 to 15% of all cancer patients [1]. This abnormal build-up of fluid in the pleural cavity frequently causes progressive shortness of breath and can be extremely debilitating for the patient [2–4]. In many cases, MPEs are a complication of end stage of cancer and significantly affects the patient’s quality of life.

Current clinical management of a MPE is palliative and involves drainage of the accumulated fluid to relieve discomfort, frequently followed by a procedure termed pleurodesis to induce obliteration of the potential pleural space through inflammation and adhesion formation [2, 3]. One of the simplest and least invasive procedures is bedside talc pleurodesis, whereby a talc slurry is instilled into the pleural cavity through a chest tube. Although usually successful, failures are known to occur for several reasons. For example, talc pleurodesis is frequently ineffective if there is incomplete expansion of the lung. In cases of complete expansion of the lung, talc pleurodesis is effective in 70 to 95% of cases, with some failures due to poor talc distribution or contact time. Foam is advantageous in increasing both the distribution of talc within the cavity and contact time between talc and tissue.

In an attempt to improve outcomes with bedside talc pleurodesis, we have developed a novel foam delivery system which showed promise in an earlier pilot study [4]. This system fills the thoracic cavity with a novel foam containing talc. The foam is formulated such that when the cold foam contacts warm tissue, the liquid fraction gels, sticks to the tissues; ultimately prolonging the contact time between talc and tissue. This is hypothesized to generate increased inflammatory response and better formation of adhesion across the pleural space.

Our previous work has evaluated the efficacy of a hydrogel poloxamer foam in enhancing talc pleurodesis in experimental models. In a rabbit model of pleurodesis, we demonstrated significant efficacy, but did not explore the relationship between the physical properties of the foam and the outcomes. We also investigated using a foam formulation as a drug delivery of talc in a mouse model of malignant pleural effusions. Talc foam improved the survival and preservation of lung volumes in this model [5] . Trials of sclerosing agents in the rabbit model of pleurodesis are well accepted as they have been shown to correlate well with clinical trials [6].

In this new manuscript, we present rheology, foam stability, and ex-vivo coating and bio-adhesion studies on a range of formulations to understand the fluid mechanics and phase transitions that would occur at physiological conditions. We then utilized an experimental rabbit pleurodesis model to evaluate the efficacy of select foam formulations.

## Methods

### Solution preparation

A novel triblock copolymer (TCH) composed of poly (oxyethylene) -poly (oxypropylene)-poly (oxyethylene) in purified form (TDLI, Princeton, NJ) was used with sterile normal saline solutions for animal experiments and for bench top and ex vivo experiments. Talc (Sigma-Aldrich, 350 mesh) was used throughout. The composition of the formulations TF-15, TF-20, and TF-22.5, TF-25 varied by concentration and are listed in Table 1. For animal experiments, the solutions were prepared in a sterile fashion.

**Table 1:** Compositions of foaming solutions

	Triblock Copolymer Hydrogel (TCH) Solution		
Formulation	Normal Saline (wt%)	TCH (wt%)	Talc (wt%)
TF-15	85	15	25
TF-20	80	20	25
TF-22.5	77.5	22.5	25
TF-25	75	25	25

### Rheology

Rheological studies of the formulations were performed on a DHR-3 rheometer, TA Instruments (New Castle, DE). All rheological experiments were performed using parallel plate of 40 mm in diameter with a gap of 1 mm. A Peltier system was used to perform temperature ramps by heating the bottom plate. All data was collected in the linear regime as confirmed by amplitude sweeps. A thin layer of silicone oil was added surrounding the plates to prevent evaporation during the experiment. A heating rate of 1.45 °C/min was utilized. The complex modulus, G*, storage modulus, G’, and loss modulus G", were measured for each of the formulations.

The thermal transition from sol to gel measured by small angle neutron scattering, was shown to correspond qualitatively to the derivative of complex modulus with respect to temperature [7]. We define this temperature as the gel phase temperature, ${T}_{gp}=T\left({\left.\frac{\partial {G}^{\ast }}{\partial T}\right\Vert}_{max}\right)$. In most cases, this simultaneously corresponds to the temperature at which G' is about an order of magnitude greater than G''. Note that T_gp_ is different than the critical gel temperature, T_gel_, which usually refers to a critical point between the isotropic phase and the gel phase. The results are presented in terms of T_gp_ since T_gel_ can vary depending on sample preparation.

### Foam formation

Foams were formed by adding 100 mL (animal model and vertical flow experiments) or 250 mL (foam persistence) of solution into a 500 ml commercial whipped cream canister (Stainless Steel Professional Cream Whipper, Tessor) charged with 8 g nitrous oxide (N_2_O) and continuously mixed prior to dispensing with a magnetic stirbar. For the foam persistence experiments, foam was discharged from the canister through an adult chest tube. For the animal model and vertical flow test, the foam was first ejected into a beaker, loaded into a syringe, and administered through a 6 French pediatric feeding tube.

### Foam persistence

The foam volume was measured from digital images taken at regular intervals of foam dispensed into a 1 L glass beaker maintained at 37 °C in a water bath. Foam volume, *V*_*f*_, was defined as the total foam volume minus the original liquid volume. The percent persistence of the foam, *P*, is defined as *V*_*f*_ normalized by the initial foam volume, *V*_*fi*_, i.e.

1 $$ P=\frac{V_f}{V_{f_i}}\times 100,\kern0.5em $$

### Animal model

The animal model is based on a well-described rabbit model of pleural adhesions [4, 8]. Briefly, New Zealand white male rabbits weighing 2.0 to 3.0 kg were utilized. A modified 6 French pediatric feeding tube was tunneled and introduced into the right pleural space. A 3 ml test liquid “agent” was injected into the right pleural space. The air was drawn out of the cavity to re-inflate the lung. The tube was left in place until the drainage was minimal. The rabbits were recovered and sacrificed after 28 days and their thoraces removed in bloc and preserved. Animal studies were approved by the Drexel IACUC and performed under the standards of the Guide for the Care and Use of Laboratory Animals. Pleurodesis was performed on 36 rabbits, TF-22.5 and TF-20 were tested on 10 rabbits each, while TF-15 was tested on 9 rabbits. The TS control was tested on the remaining 7 rabbits. Additional results for TS, NS, and Foam controls were pooled from a previous study [4].

### Pathology

**Fig. 1:**
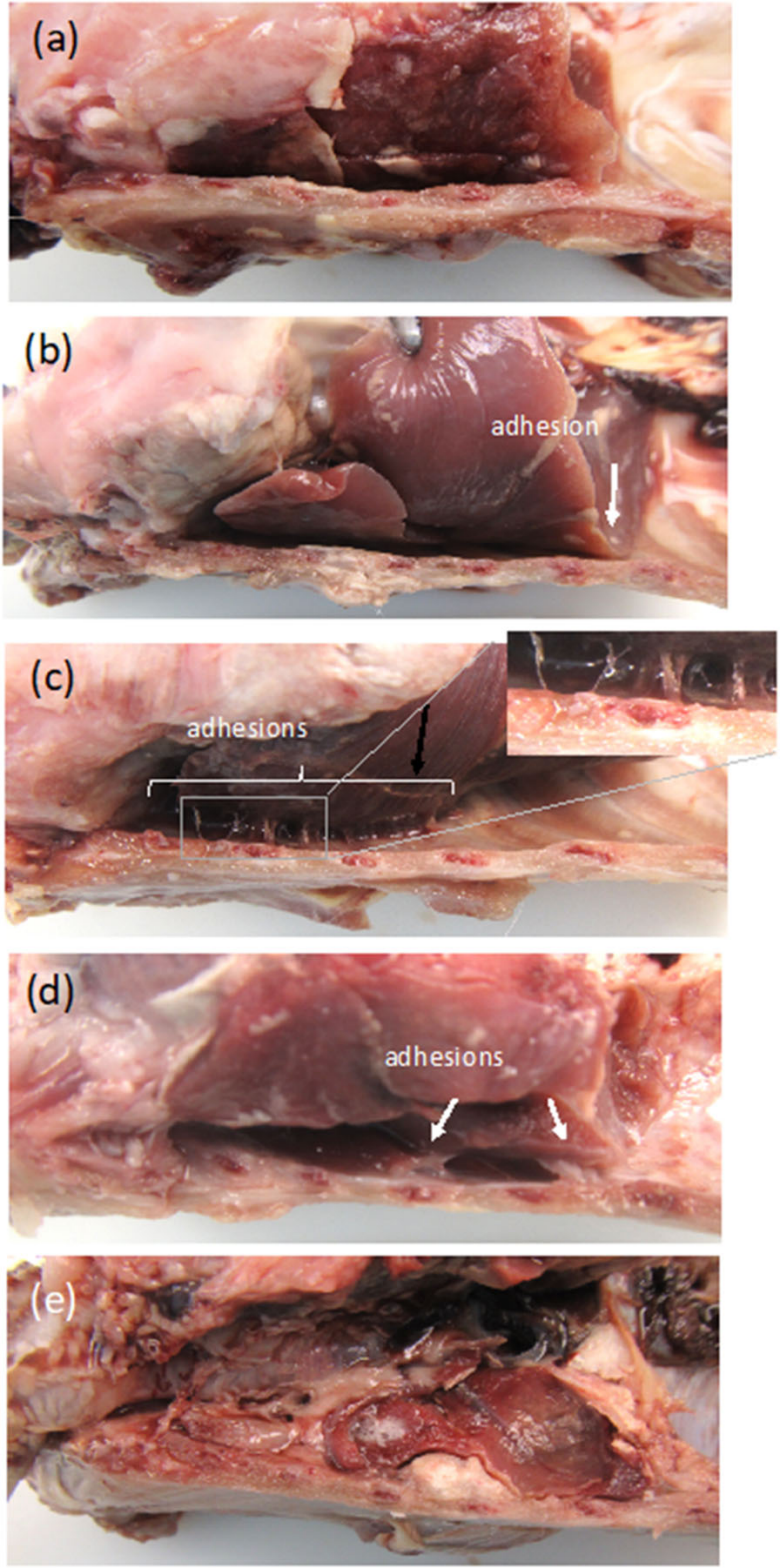
Scoring system for adhesions: Thorax sections of rabbits with the amount of adhesions between the lung and chest wall scored (**a**) Score 0: No adhesions between lung and chest wall. (**b**) Score 1: A few adhesions. (**c**) Score 2: Scattered adhesions. (**d**) Score 3: Generalized scattered adhesions. (**e**) Score 4: Complete obliteration of the pleural space

The degree of pleurodesis was evaluated according to an established scoring system by a blinded pathologist and graded: 0: normal pleural space; 1: one to three small adhesions in the pleural space; 2: more than three scattered adhesions with lung easily separated from the chest wall; 3: generalized scattered adhesions with areas where the lung can be separated from the chest wall only with difficulty; and 4: complete obliteration of the pleural space (Fig. 1) [8].

### Vertical flow test

Food grade porcine ribs were used as a model of pleural surface. Foam was delivered onto moistened horizontal ribs incubated to 37 °C at a temperature-controlled room, in a similar manner as the animal study. Within a minute, each rib sample was hung vertically to assess foam runoff and quality of coating. The samples were imaged sequentially and reviewed to measure the distance of foam flow and surface coating. These tests were repeated tree times to ensure the accuracy of the results.

### Statistics

The ANOVA student t-test two-sample unequal variance with two tails was performed on scores of adhesions.

## Results

**Fig. 2:**
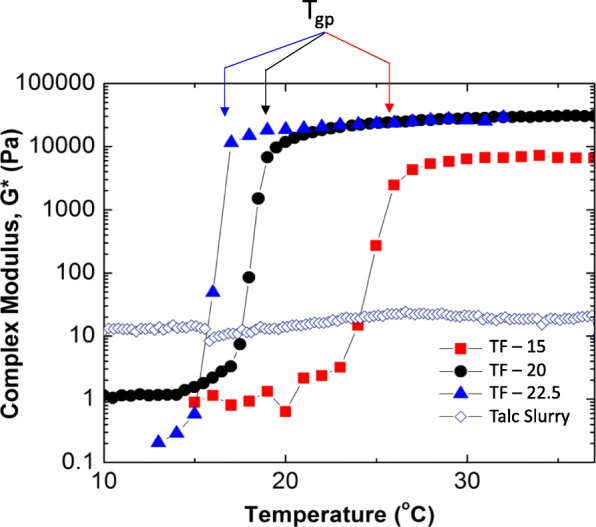
Temperature-dependent material properties of the three formulations without talc used to create the foams. The complex modulus of the talc slurry is independent of temperature. At temperatures below the step change in modulus the solutions behave as a liquid, above T_gp_ they are gels and do not flow

Three formulations of talc, normal saline and the reverse triblock copolymer hydrogel (TF-15, TF-20, and TF-22.5) were studied using small amplitude oscillatory shear rheology (SAOS) to determine their temperature-dependent complex modulus (Fig. 2). Table 2 shows T_gp_ for the three samples. At temperatures higher than T_gp_, the formulations have a complex modulus 3–4 decades higher than below T_gp_, which indicates a sol-gel phase transition [7]; i.e. a significant step change from a flowing liquid to a non-flowing gel. This behavior is in stark contrast to the talc slurry, which shows no temperature-dependent complex modulus. The onset temperature of gelation is approximated as the temperature at which the complex modulus begins to sharply increase and is generally 5 °C lower than T_gp_. Table 2 summarizes the important parameters from Fig. 2.

**Table 2:** Temperature of onset of gel phase and complex moduli at physiological temperatures of three formulations of normal saline and triblock copolymer hydrogel, and of talc slurry

	Temperature at beginning of modulus step change (°C)	T_gp_ (°C)	G* at 37 °C (Pa)
TF-15	~ 23	26	6500
TF-20	~ 17	19	30,000
TF-22.5	~ 14	17	30,000
Talc Slurry	NA	NA	20

**Fig. 3:**
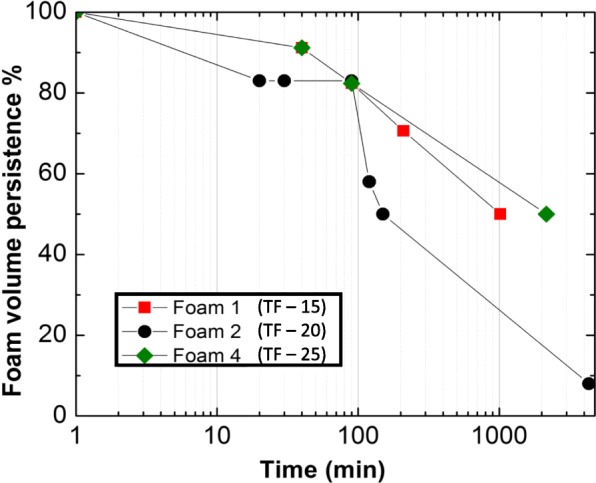
Foam persistence measurements performed in vitro. Approximately 1 L of foam, dispensed at low temperature, was placed in a glass beaker in a 37 °C water bath and imaged over time

The length of time in which the volume of foam persists (i.e. does not collapse) may be important to the efficacy of pleurodesis by foam. One hypothesis for increased efficacy of pleurodesis with talc delivered with foam [4] is that the increased volume of foam distributes talc over a larger surface area. The persistence of the foams was tested in vitro within a glass container at 37 °C designed to mimic foam delivered into the pleural cavity at 37 °C. Foam persistence measurements were made by foaming three formulations (TF-15, TF-20, and TF-22.5) to volumes 3.6, 3.8, and 3.5 (stdev ±0.6, 0.4, 0.4 (*n* = 10)) times the solution volume, respectively. Figure 3 shows the percent persistence as a function of time. All three foams persisted to *P* = 80% for 100 min. At two hours, i.e. the usual time that the talc slurry is drained during pleurodesis, showed *P* > 50% for all three formulations.

**Fig. 4:**
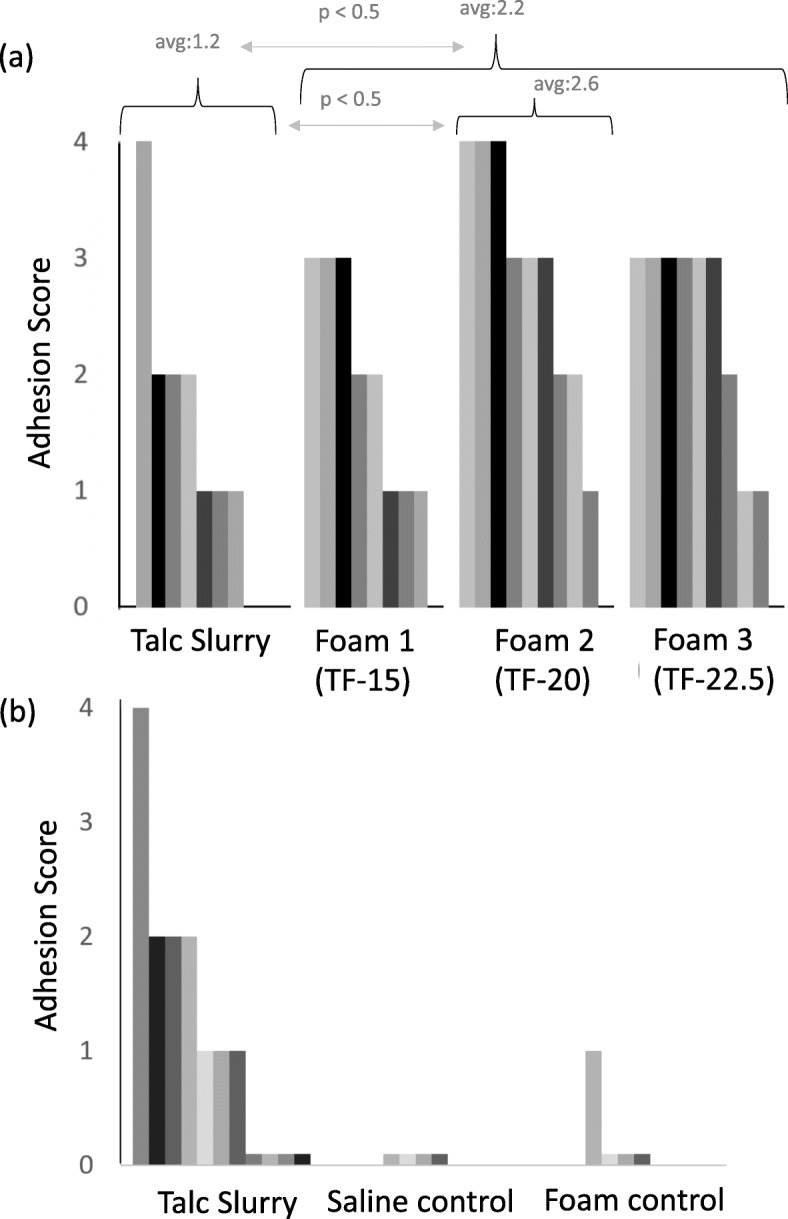
The effectiveness of pleurodesis by talc delivered by foam or slurry was assessed by the degree of adhesions formed after 28 days in a rabbit model. (**a**) The delivery of talc with foam significantly increased the degree of adhesions (2.2 vs. 1.2, *p* < 0.5). Foam 2 produced adhesions that were significantly greater that delivery by slurry (2.6 vs 2.2, *p* < 0.05). (**b**) The method of talc delivery rather than the formulations in the absence of talc were responsible for the adhesions

The effectiveness of pleurodesis with talc delivered by foam using TF-15, TF-20, and TF-22.5 was assessed using our rabbit model. The efficacy was quantified utilizing a numerical score that incorporates the number and completeness of adhesions formed within the pleural cavity after 28 days, see Fig. 4a. Previously, adhesions caused by delivery of talc by slurry (Talc + Saline: 1 g of talc [10 μm, Sigma] which was mixed with 3 mL of normal saline by shuttling between two syringes just prior to delivery), was evaluated according to the same methods and used to compare the results of this study [4]. Delivery of talc to the pleural cavity by the foam (data of all three formulations shown in Fig. 4a) resulted in significantly greater adhesion formation than delivery by talc slurry as shown in Fig. 4b (adhesion score: 2.2 vs 1.2 *p* < 0.05). One formulation, Foam 2, outperformed the other two when compared to talc slurry (2.6 vs 1.2, *p* = 0.02). Figure 4b also shows that the hydrogel foam without talc and saline controls resulted in minimal formation of adhesions, confirming the importance of talc in the process.

**Fig. 5:**
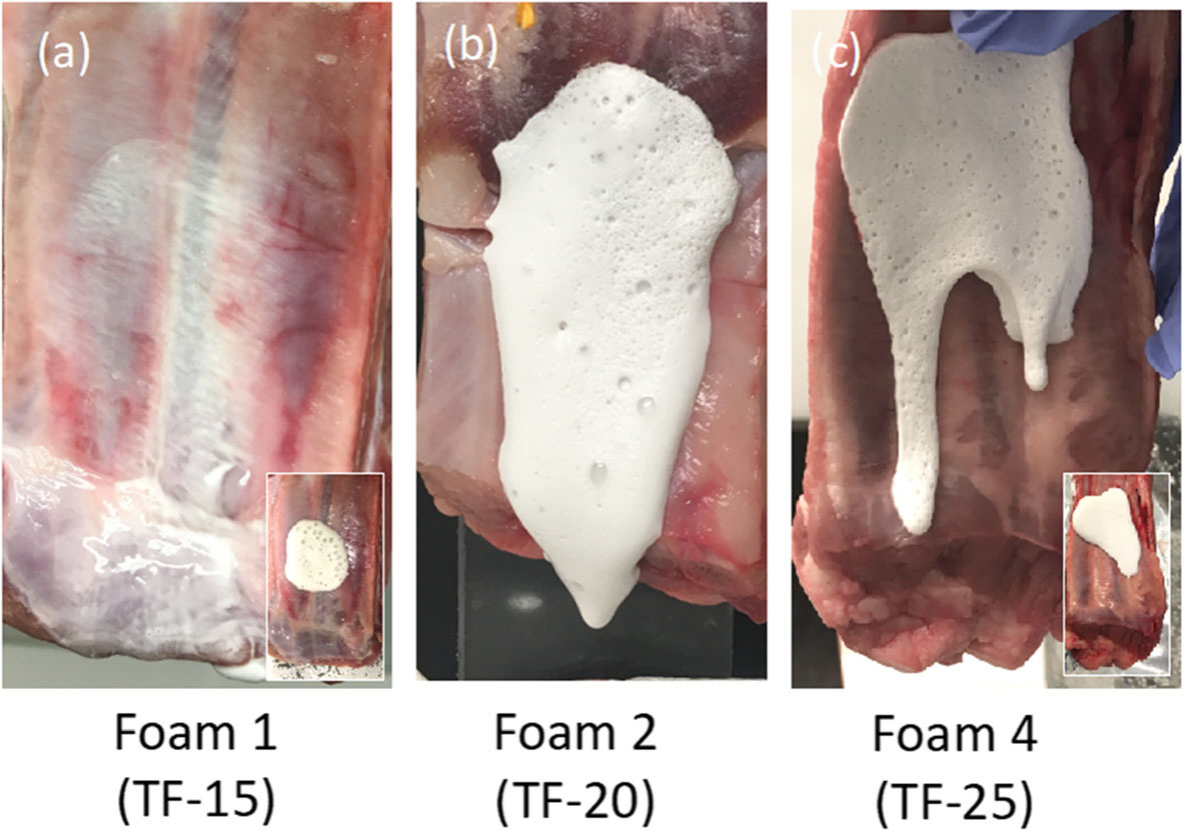
Vertical Flow Test of Foams 1, 2, and 4 on porcine chest walls ex vivo at 37 °C. The insets show the original areas of foam delivery, applied horizontally, prior to tissue being hung vertically. (**a**) Foam 1, created from TF-15, flowed off the tissue leaving residual solution behind. (**b**) Foam 2, created from TF-20, flowed down the tissue leaving gelled foam coating the tissue. An image of the original area of administration was not available but was similar to the inset of (**a**). (**c**) Foam 4, created from TF-25, mostly remained in the original location of delivery with the exception of a few isolated drips

To evaluate the coating properties of the foam on parietal pleura, we developed the Vertical Flow Test model. In this model we utilized porcine ribs to serve as a model for the chest wall. The temperature of the ribs and the surrounding environment was kept at physiological temperatures. The foams were delivered to the tissue in the same manner as was performed in the in vivo animal model (Fig. 5). Initially the foams were deposited onto the porcine rib specimens with the ribs laying horizontal with respect to gravity and then were hung vertically. The original area of foam coverage is noted in the insets in (a) and (c). The original area of foam coverage of (b) was similar to that of (a). From the Vertical Flow Test, TF-20 foam coated the largest area of chest wall (Fig. 5b). The TF-25 foam rapidly gelled in the location it was delivered, resulting in limited dispersion and less coverage of the area than TF-20 (Fig. 5c). TF-15 when foamed, flowed off the tissue leaving behind some talc residue (see white region in Fig. 5a). The gelation of all delivered foam is apparent during the experiment when the foam abruptly stops flowing. The samples were monitored for an additional 10 min and no changes were observed with time; demonstrating that Fig. 5 represents a steady state.

## Discussion

Our results demonstrate that delivery of talc by foam significantly increases the number of adhesions in a rabbit model compared to the current treatment using talc slurry. This result was anticipated given the results of a previous study [4]. However, this study also suggests that there is an optimum concentration of the reverse triblock copolymer hydrogel. The rheological investigations found that TF-22.5 had the lowest T_gp_ at 17 °C compared with 26 and 19 °C for TF-15 and TF-20, respectively. Delivery of foams ex vivo to porcine chest walls shows that delivery of a cold foam, with a low T_gp,_ warms above T_gp_ the fastest. Furthermore, the hydrogel control in Fig. 4b confirms that the hydrogel itself played no active role in adhesion formation. These results suggest there is an optimum time to reach gelation inside the pleural cavity that gives the most homogenous talc deposition.

The development of a clinically useful temperature-sensitive foam for talc delivery needs to consider the anticipated delivery process. The foam should flow easily in the liquid state through the delivery tube and into the 37 °C pleural cavity where it should homogeneously coat and gel on the tissue walls. Heat transfer from the body increases the temperature of the foam starting in the delivery tube and inside the pleural cavity. For proper delivery, the temperature of the foam must remain below T_gp_ for the entire delivery step to prevent clogging the tube. Once delivered to the pleural cavity, the time to reach T_gp_ is expected to determine the type of foam coverage. One can consider two situations where the time to reach T_gp_ can lead to nonuniform foam delivery to the pleural cavity: [1] if the foam collapses before gelation and [2] if the foam reaches T_gp_ and gels before it fills the entire pleural cavity. There are two engineering parameters that effect the time to gelation: the initial foam temperature *T*_*i*_ and *T*_*gp*_.

In this study, TF-15 foam, TF-20 foam, and TF-22.5 foam were delivered at initial foam temperatures of *T*_*i*_= 16.8 °C, 8.6 °C, and 6.4 °C, respectively with T_gp_ values of 26, 19, and 17 °C, respectively. Note that all solutions have approximately the same ΔT ≈ 10 °C rise required for gelation. Few side effects of the foam were seen except for a significant drop in heart rate in the rabbits after injecting the cold solution into the thoracic cavity. The effect were transient and lasted for about 30 s, after which the heart rate returned to normal.

If we assume that the material parameters such as density, heat capacity, and the heat transfer coefficient are independent of TCH concentration, then it can be shown that the time to gelation decreases with increasing TCH concentration. From first principle heat transfer, the time to reach *T*_*gp*_ is given by:

2 $$ {t}_{gel}=-{\tau}_c\ \ln \frac{T_{\mathrm{gp}}-{T}_{\mathrm{cavity}}}{T_i-{T}_{\mathrm{cavity}}} $$

where *τ*_*c*_ = *ρVC*_*p*_/(*hA*) considering convective heat transfer and *T*_cavity_ is the temperature of the body. Using this equation, TF-20 reaches gelation 1.4 times faster than TF-15 and TF-22.5 reaches gelation 1.1 times faster than TF-20. This timescale analysis is validated in the vertical flow test experiments of TF-20 foam and above, which shows that there is decreasing flow of the foam from the surface of the tissue with increasing the hydrogel concentration. In other words, TF-15 flows before it has time to gel, resulting in inhomogeneous foam coverage. TF-22.5 gels much faster and does not allow the foam to completely cover the tissue. TF-20 has an effective balance such that a moving gelation front occurs on the tissue surface: coating and gelling in the pleural cavity; leading to more uniform talc deposition and subsequently increased pleural adhesions.

Another complication with TF-15 is dilution on the tissue interface. While the TF-15 takes longer to gel, an additional set of Vertical Flow Test experiments were conducted at long incubation times (10 min) to ensure all samples had gelled before vertical inversion. TF-15 still flowed off the tissue specimen (*n* = 3); indicating poor adhesion to the tissue surface. We hypothesize that this is due to dilution near the tissue surface. The phase diagram for the given TCH indicates that a dilution of more than 1 wt% of normal saline in TF-15 would create a solution with no gel phase at 37 °C. Therefore, the amount of fluid in the cavity prior to foam delivery could be an important aspect in determining the concentration of TCH.

One important implication of these arguments is that delivery of talc with the triblock copolymer hydrogel foam is dependent on the time to reach the gelation temperature and the minimum concentration of TCH. Note that in order to increase *t*_*gel*_ of TF-20 would require a significant reduction in temperature, at which point the freezing point of the solution would need to be taken into account. In any case, the final formulation would benefit in reducing the amount of TCH introduced in vivo to minimize dosage.

## Conclusions

In previous works we demonstrated the improved efficacy of one foam formulation for pleurodesis in both mice and rabbit models. Here in, we determine the importance of the foam formulation’s physical properties via different concentrations of poloxamer on the adhesion score for pleurodesis in a rabbit model. For example, we have identified some of the rheological parameters that enhance pleural adhesions for a fixed concentration of talc. We hypothesize that the improved efficacy of TF-20 is mainly due to its optimum time to gelation. This foam delivery system has promising potential for clinical applications and will be the subject of future investigations using larger animals with pleural cavities more closely resembling patients.

## Data Availability

All data generated or analyzed during this study are included in this published article.

## Abbreviations

ANOVA: Analysis of Variance
FDA: Food and Drug Administration
MPEs: Malignant pleural effusions
TCH: Triblock copolymer hydrogel
TF: Talc foam
TF-15: Talc foam formulation with 15 wt% TCH and 29 wt% talc
TF-20: Talc foam formulation with 20 wt% TCH and 29 wt% talc
TF-22.5: Talc foam formulation with 22.5 wt% TCH and 29 wt% talc
TF-25: Talc foam formulation with 25 wt% TCH and 29 wt% talc
TS: Talc slurry
